# Clinical condition and medication therapy of amoxicillin‐induced Stevens‐Johnson syndrome: A case report

**DOI:** 10.1002/agm2.12088

**Published:** 2019-11-14

**Authors:** Sivannan Srinivasan, Elumalai Karthikeyan, Srinivasan Sivaneswari, Eluri Kalpana, Elumalai Manogaran, Krishnan Karthickeyan

**Affiliations:** ^1^ Department of Pharmacy Practice J.K.K. Nataraja College of Pharmacy Komarapalayam India; ^2^ Department of Pharmacy Practice Karpagam College of Pharmacy Coimbatore India; ^3^ Department of Medicinal Chemistry Seven Hills College of Pharmacy Tirupati India; ^4^ Department of Pharmaceutics K.K. College of Pharmacy Chennai India; ^5^ Faculty of Pharmaceutical Sciences UCSI University Kuala Lumpur Malaysia; ^6^ Department of Pharmacy Practice SRM College of Pharmacy Kattankulathur India

**Keywords:** amoxicillin, Naranjo's Adverse Drug Reaction Probability Scale, prednisolone, Stevens‐Johnson syndrome

## 1 INTRODUCTION

Cutaneous emission is a regular type of unfavorable medication response. Stevens‐Johnson syndrome (SJS) is one such example of a serious cutaneous emission. The condition presents with extreme purulent conjunctivitis, serious stomatitis with broad mucosal putrefaction, and purpuric macules. The etiology is connected to the utilization of medications as opposed to other factors.^1^ The normal guilty parties are antimicrobials, such as sulfonamide, followed by nonsteroidal  anti‐inflammatory drugs (NSAIDs), anticonvulsant medications, and tranquilizers used to treat gout. Here, we report an instance of SJS instigated as a result of an overdose of amoxicillin.^2^

## 2 CASE REPORT

A 63‐year‐old male patient presented to the medical clinic with grievances of rashes everywhere throughout the body and sores around the lips since 3 days. The patient had a history of medication ingestion for fever and after ingestion of the medication, injuries appeared on his lips within 12 hours and the seriousness of the response was noted within 24 hours. On examination, vesicular sores over the tongue, buccal mucosa, and lips were noted and the fundamental examination generally revealed no other critical discoveries. On detailed enquiry, the patient gave a background marked by ingestion of amoxicillin 500 mg, after which he experienced swelling over the lips and rashes over the body. The patient's history revealed that he was not hypersensitive to any sort of medications and neither was his family.

His research center esteem demonstrated huge increment in differentially (polymorphs: 72%; eosinophil: 12%). Considering his history, clinical examination, and research facility discoveries, the patient was diagnosed as having amoxicillin‐induced SJS. We used Naranjo's Adverse Drug Reaction Probability Scale and the World Health Organization (WHO) Causality Scale to determine whether SJS could have been caused by amoxicillin as an adverse drug reaction (ADR). The Naranjo score for the suspected ADR was 7 and thus SJS was a likely ADR presumably brought about by the amoxicillin (Table 1). The total rating of the suspected ADR with the WHO Causality Scale indicated that SJS was a likely ADR brought about by the amoxicillin it is mentioned in Table 2.

**Table 1:** Naranjo's Adverse Drug Reaction Probability Scale

No.	Question	Yes	No	Don't know
1.	Are there previous conclusive reports on this reaction?	**+1**	0	0
2.	Did the adverse event appear after the suspected drug was given?	**+2**	−1	0
3.	Did the adverse reaction improve when the drug was discontinued or a specific antagonist was given?	**+1**	0	0
4.	Did the adverse reaction appear when the drug was re‐administered?	+2	−1	**0**
5.	Are there alternative causes that could have caused the reaction?	−1	**+2**	0
6.	Did the reaction reappear when a placebo was given?	−1	+1	**0**
7.	Was the drug detected in any body fluid in toxic concentrations?	+1	**0**	0
8.	Was the reaction more severe when the dose was increased, or less severe when the dose was decreased?	**+1**	0	0
9.	Did the patient have a similar reaction to the same or similar drugs in any previous exposure?	+1	**0**	0

Scoring Scale: >9 = definite ADR, 5‐8 = probable ADR, 1‐4 = possible ADR, 0 = doubtful ADR.

Score: 7.

Result: Our case's total Naranjo score for the suspected ADR was 7; therefore, Stevens‐Johnson syndrome was a probable ADR caused by the suspected drug amoxicillin. Bold values indicate the obtained result.

**Table 2:** World Health Organization Causality Scale

No	Causality term	Assessment criteria
1.	Certain	Event or laboratory test abnormality, with plausible time relationship to drug intake Cannot be explained by disease or other drugs Response to withdrawal plausible (pharmacologically, pathologically) Event definitive pharmacologically or phenomenological (ie, an objective and specific medical disorder or a recognized pharmacologic phenomenon) Rechallenge satisfactory, if necessary
2.	Probable/likely	Event or laboratory test abnormality, with reasonable time relationship to drug intake Unlikely to be attributed to disease or other drugs Response to withdrawal clinically reasonable Rechallenge not required
3.	Possible	Event or laboratory test abnormality, with reasonable time relationship to drug intake Could also be explained by disease or other drugs Information on drug withdrawal may be lacking or unclear
4.	Unlikely	Event or laboratory test abnormality, with a time to drug intake that makes a relationship improbable (but not impossible) Disease or other drugs provide plausible explanation
5.	Conditional/unclassified	Event or laboratory test abnormality More data for proper assessment needed Additional data under examination
6.	Unassessable/unclassifiable	Report suggesting an adverse reaction Cannot be judged because information is insufficient or contradictory Data cannot be supplemented or verified

Result: Our case's total rating of the suspected ADR with the WHO Causality Scale categorized Stevens‐Johnson syndrome as a probable ADR caused by the suspected drug amoxicillin.

The patient was treated with parenteral antimicrobial (Inj. cefotaxime 1 g, bd), antibiotic (T. Chloramphenicol maleate 4 mg, bd), treatment of injuries (Oint. triamcinolone acetonide buccal glue 0.1%), and steroids beginning from the third day (T. prednisolone 5 mg, od). An alarm card for the suspected ADR was given to the patient by the Drug Information Center, Government Headquarters' Hospital, Erode, India, for future awareness.

## 3 DISCUSSION

In 1922, Stevens and Johnson depicted two male patients of 7 and 8 years of age who experienced unprecedented summed up ejection with fever and inflamed buccal mucosa.^2^ SJS can be separated from other skin conditions according to three clinical criteria: (1) individual skin injuries, (2) appropriation of sores, and (3) degree of epidermal separation. The trademark discoveries in SJS are broad erythematous or purpuric macules that structure level atypical target injuries as the sickness advances to cause full‐thickness epithelial necrosis.^3^

In the oral cavity, SJS causes across‐the‐board ulcerative sores. A prodrome happens in around 30% of cases and may start within 1 to 3 weeks of beginning another medication and endures 1 to about 14 days before the beginning of mucocutaneous appearances, giving influenza‐like indications, sore throat, migraine, arthralgia, myalgia, fever, bullous and different rashes, pneumonia, nephritis, or myocarditis.^4^ Balanitis, urethritis, and vulvar ulcers may also occur. Our patient did not report any prodrome, yet skin, mouth, and genital ulcerations were present. Medication‐induced SJS is portrayed by mucosal disintegrations in addition to far‐reaching circulation of atypical targets or purpuric macules and epithelial separation, including under 10% body surface area on the storage compartment, face and extremities.^1^ SJS must be clinically separated from viral stomatitis, pemphigus, erythema multiforme (EM), toxic epidermal necrolysis, and the subepithelial resistant rankling issue, such as pemphigoid. There are no particular demonstrative tests for SJS.^5^ Our case indicated ulceration of the oral cavity, eye redness, ulceration of genital locale alongside various recuperated injuries on the chest, midriff, and appendages, which indicated ordinary appearance of “target sores.” The sores were across the board when contrasted with EM, which is confined.

Amoxicillin and clavulanic corrosive mix treatment was recognized as the causative specialist in light of the fleeting connection between the organization of the mix and the start of the ejections. There have likewise been a few different past reports connecting amoxicillin and clavulanic corrosive to SJS. As per Naranjo's Adverse Drug Reaction Probability Scale, amoxicillin‐induced SJS was conceivable in our patient (a score of 7). The initial phase in the administration was a quick withdrawal of the culpable operator followed by strong consideration. A past report reveals that a prompt withdrawal of the medication may reduce the mortality risk.^5^ Adjuvant medicines, for example, corticosteroids, may be utilized in extreme instances of SJS.^6^

## 4 CONCLUSION

In developing nations like India, where unavoidable illnesses are generally predominant, anti‐infection agents ought to be utilized with caution to avoid ADR. Overuse of medications such as NSAIDs, anti‐infection agents, and anti‐seizure drugs can result in SJS and dangerous epidermal necrolysis, which is a perilous condition. Medical practitioners should be aware of the risks and advise their patients accordingly. Amoxicillin‐induced SJS can be treated with anti‐infection agents, such as cefotaxime and chloramphenicol maleate, and with corticosteroids. A hazard evaluation is required for avoiding harm to extra tissue.

## CONFLICTS OF INTEREST

There are no conflicts of interest.

## AUTHOR CONTRIBUTIONS

We declare that this work was done by the authors named in this article. E. Karthikeyan, M.E., and S. Srinivasan conceived and designed the study. S.S. carried out the laboratory work. K.K., S. Sivaneswari, and E. Kalpana analyzed the data and wrote the manuscript. All authors have read and approved the final manuscript.
